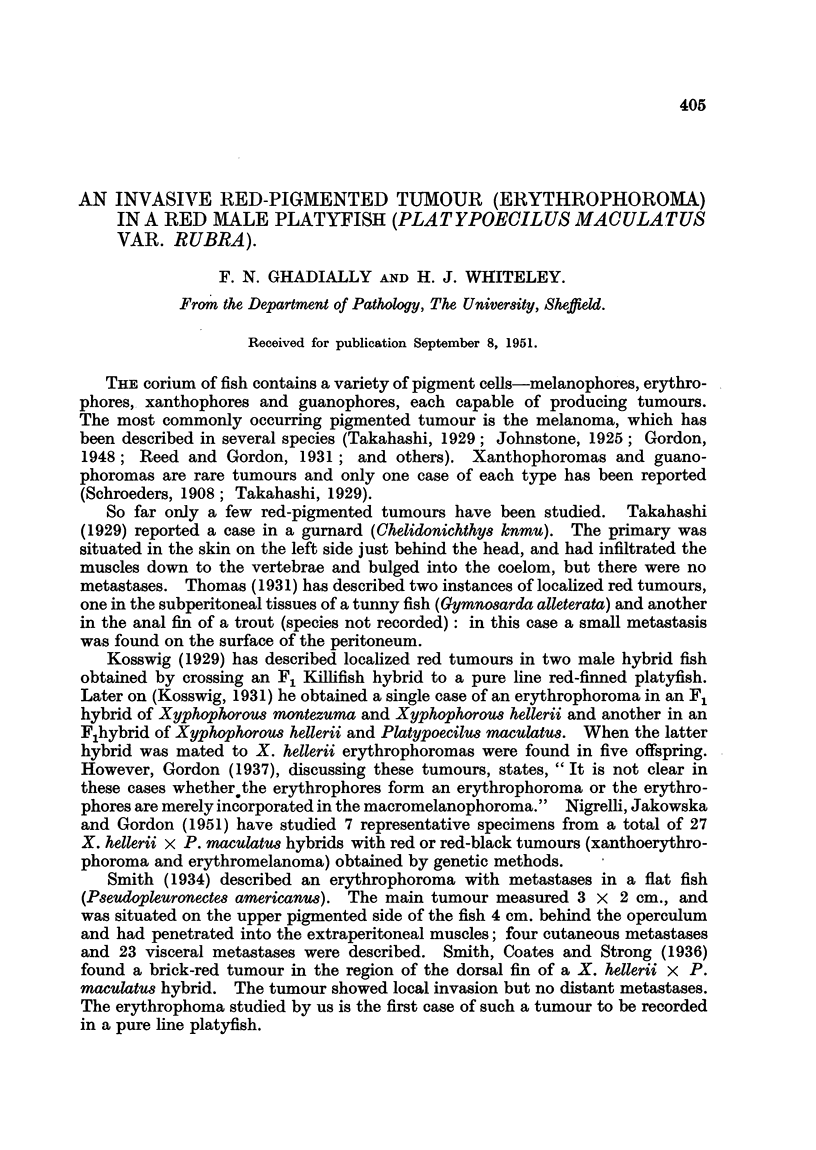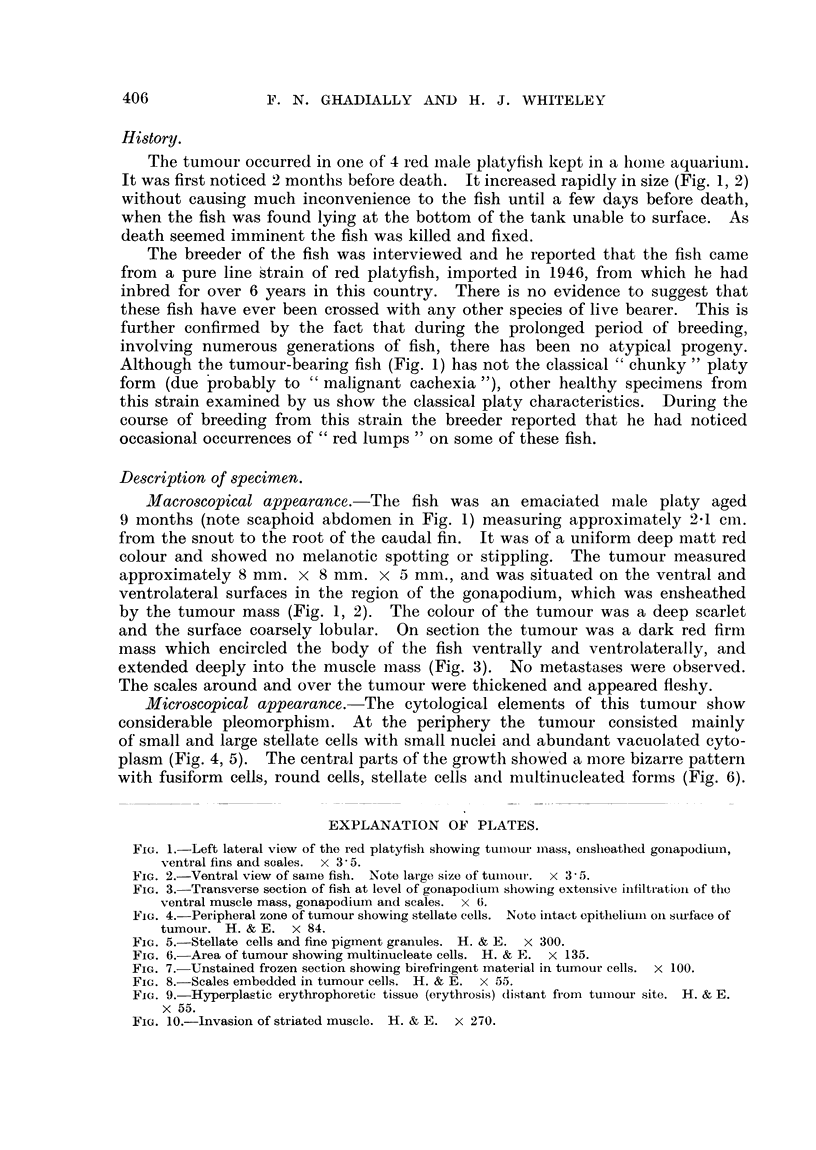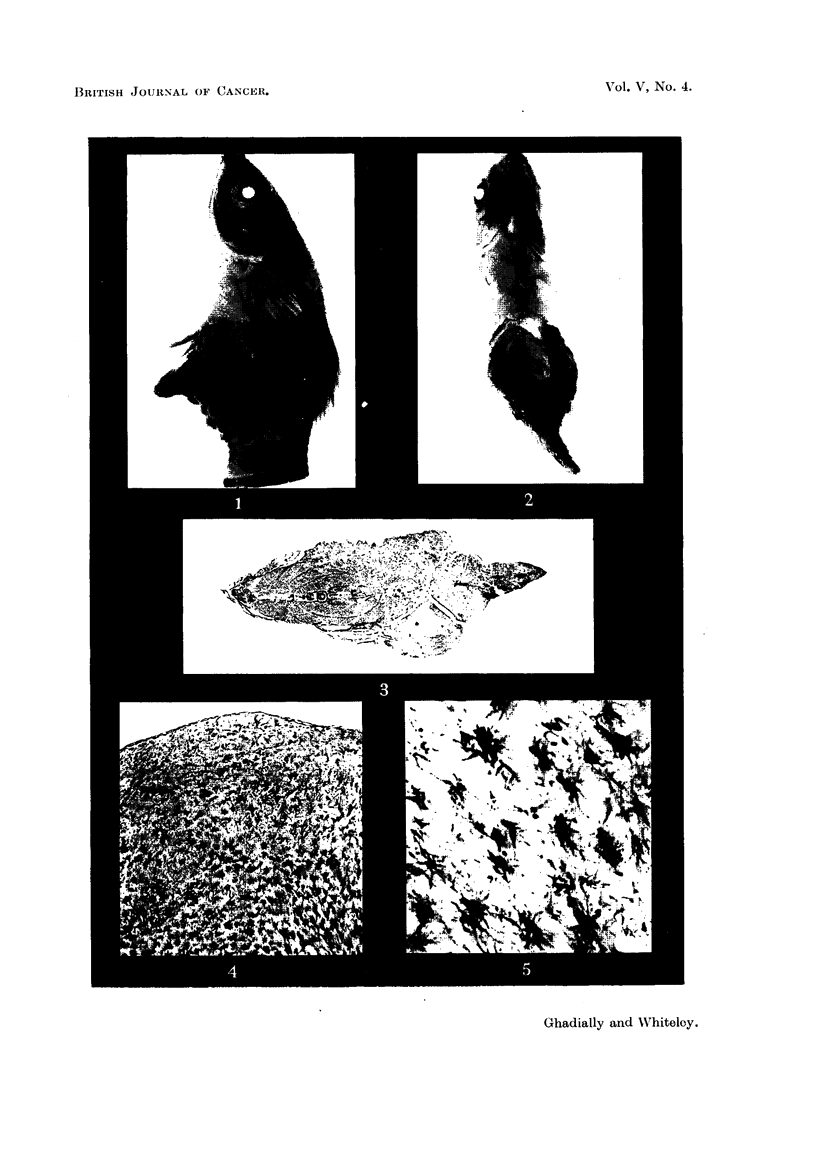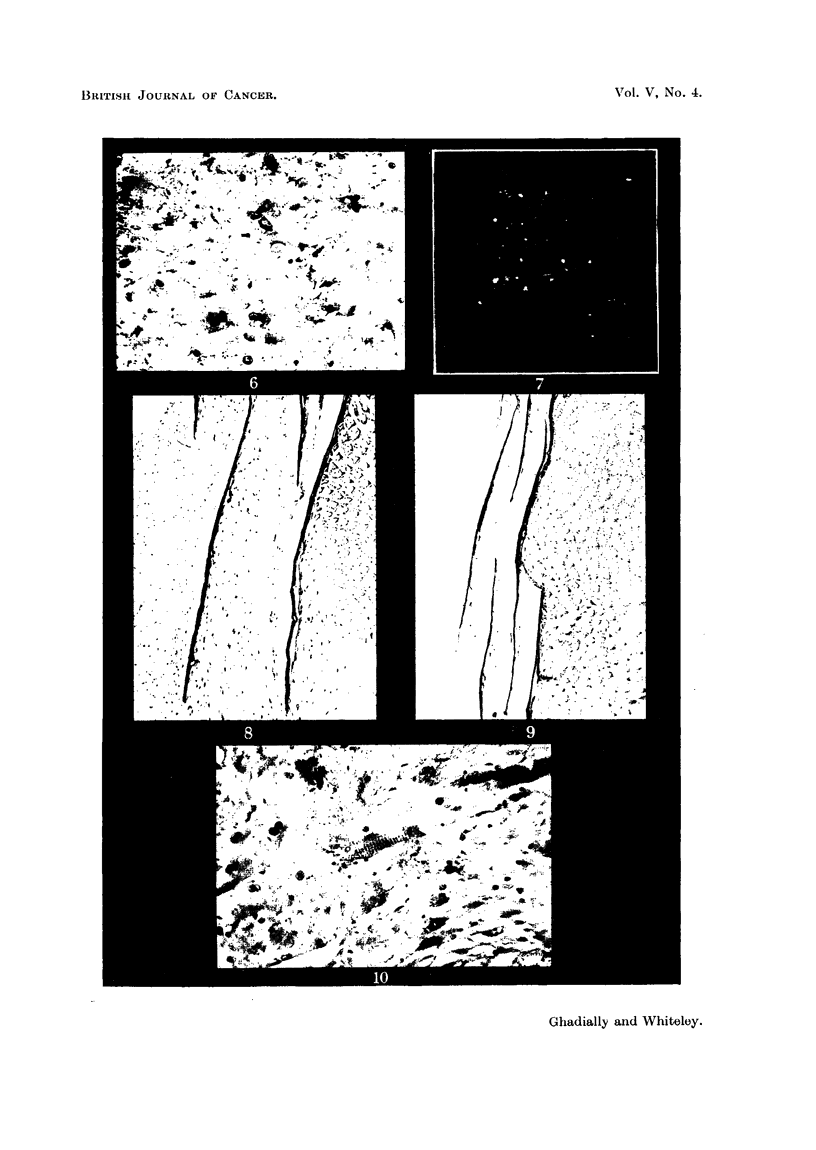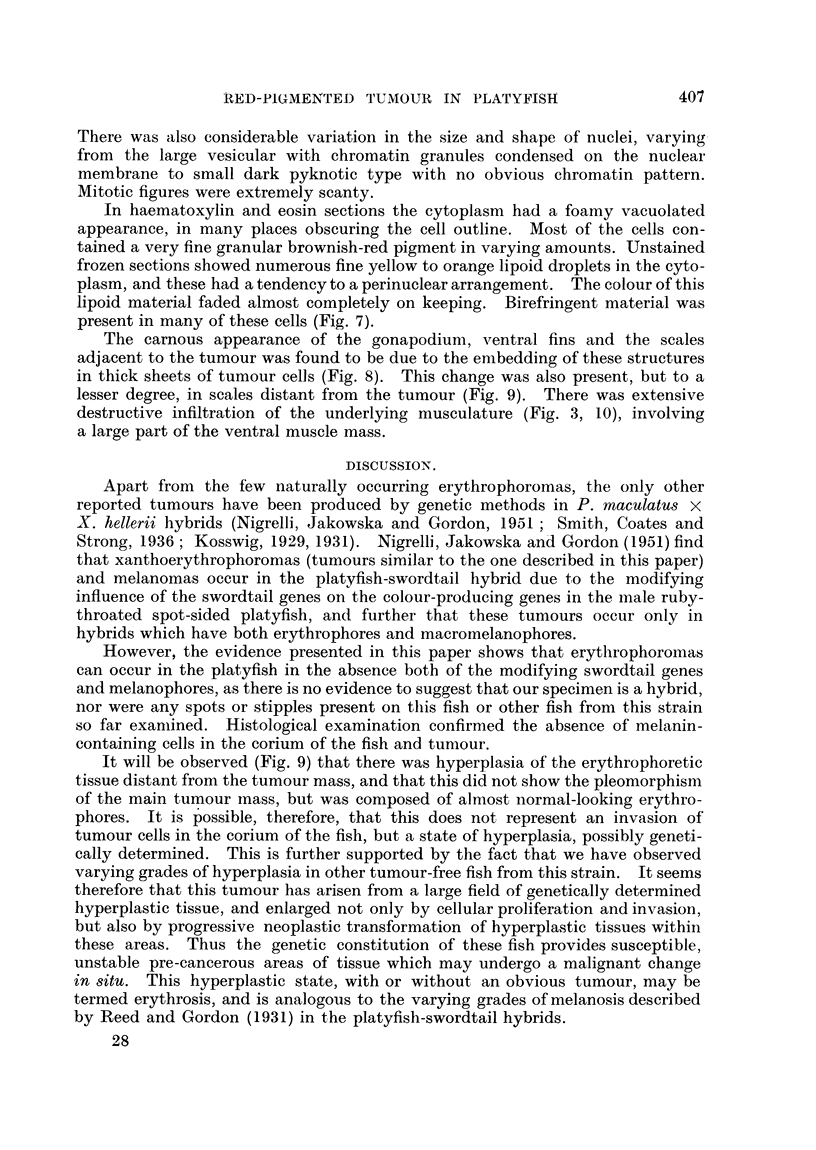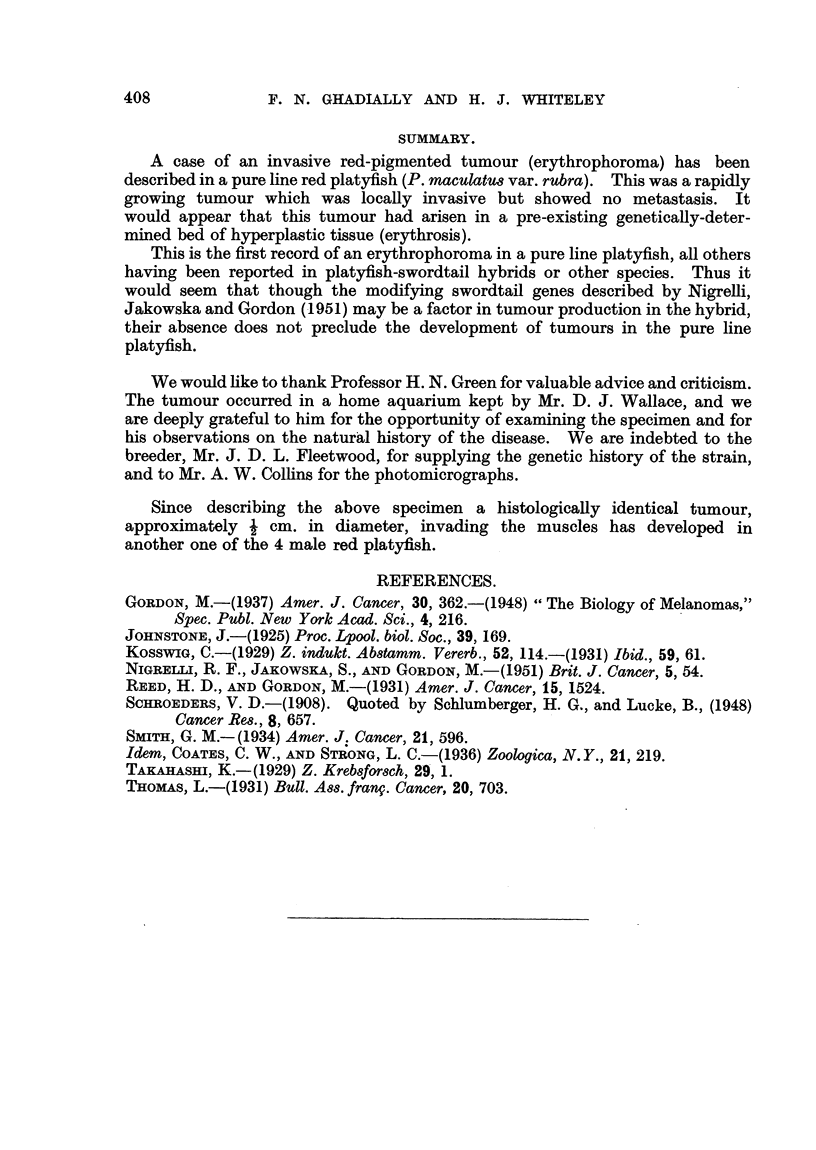# An Invasive Red-Pigmented Tumour (Erythrophoroma) in a Red Male Platyfish (Platypoecilus Maculatus var. Rubra

**DOI:** 10.1038/bjc.1951.46

**Published:** 1951-12

**Authors:** F. N. Ghadially, H. J. Whiteley

## Abstract

**Images:**


### AN INVASIVE RED-PIGMENTED TUMOUR (ERYTHROPHOROMA) IN A RED MALE PLATYFISH (*PLATYPOECILUS MACULATUS* VAR. *RUBRA*).

F. N. GHADIALLY AND H. J. WHITELEY.

*From the Department of Pathology, The University, Sheffield.*

Received for publication September 8, 1951.

THE corium of fish contains a variety of pigment cells—melanophores, erythrophores, xanthophores and guanophores, each capable of producing tumours. The most commonly occurring pigmented tumour is the melanoma, which has been described in several species (Takahashi, 1929; Johnstone, 1925; Gordon, 1948; Reed and Gordon, 1931; and others). Xanthophoromas and guanophoromas are rare tumours and only one case of each type has been reported (Schroeders, 1908; Takahashi, 1929).

So far only a few red-pigmented tumours have been studied. Takahashi (1929) reported a case in a gurnard (*Chelidonichthys knmu*). The primary was situated in the skin on the left side just behind the head, and had infiltrated the muscles down to the vertebrae and bulged into the coelom, but there were no metastases. Thomas (1931) has described two instances of localized red tumours, one in the subperitoneal tissues of a tunny fish (*Gymnosarda alleterata*) and another in the anal fin of a trout (species not recorded): in this case a small metastasis was found on the surface of the peritoneum.

Kosswig (1929) has described localized red tumours in two male hybrid fish obtained by crossing an $F_1$ Killifish hybrid to a pure line red-finned platyfish. Later on (Kosswig, 1931) he obtained a single case of an erythrophoroma in an $F_1$ hybrid of *Xyphophorous montezuma* and *Xyphophorous hellerii* and another in an $F_1$hybrid of *Xyphophorous hellerii* and *Platypoecilus maculatus*. When the latter hybrid was mated to *X. hellerii* erythrophoromas were found in five offspring. However, Gordon (1937), discussing these tumours, states, "It is not clear in these cases whether the erythrophores form an erythrophoroma or the erythrophores are merely incorporated in the macromelanophoroma." Nigrelli, Jakowska and Gordon (1951) have studied 7 representative specimens from a total of 27 *X. hellerii* × *P. maculatus* hybrids with red or red-black tumours (xanthoerythrophoroma and erythromelanoma) obtained by genetic methods.

Smith (1934) described an erythrophoroma with metastases in a flat fish (*Pseudopleuronectes americanus*). The main tumour measured 3 × 2 cm., and was situated on the upper pigmented side of the fish 4 cm. behind the operculum and had penetrated into the extraperitoneal muscles; four cutaneous metastases and 23 visceral metastases were described. Smith, Coates and Strong (1936) found a brick-red tumour in the region of the dorsal fin of a *X. hellerii* × *P. maculatus* hybrid. The tumour showed local invasion but no distant metastases. The erythrophoma studied by us is the first case of such a tumour to be recorded in a pure line platyfish.

*History.*

The tumour occurred in one of 4 red male platyfish kept in a home aquarium. It was first noticed 2 months before death. It increased rapidly in size (Fig. 1, 2) without causing much inconvenience to the fish until a few days before death, when the fish was found lying at the bottom of the tank unable to surface. As death seemed imminent the fish was killed and fixed.

The breeder of the fish was interviewed and he reported that the fish came from a pure line strain of red platyfish, imported in 1946, from which he had inbred for over 6 years in this country. There is no evidence to suggest that these fish have ever been crossed with any other species of live bearer. This is further confirmed by the fact that during the prolonged period of breeding, involving numerous generations of fish, there has been no atypical progeny. Although the tumour-bearing fish (Fig. 1) has not the classical " chunky " platy form (due probably to " malignant cachexia "), other healthy specimens from this strain examined by us show the classical platy characteristics. During the course of breeding from this strain the breeder reported that he had noticed occasional occurrences of " red lumps " on some of these fish.

*Description of specimen.*

*Macroscopical appearance.*—The fish was an emaciated male platy aged 9 months (note scaphoid abdomen in Fig. 1) measuring approximately 2·1 cm. from the snout to the root of the caudal fin. It was of a uniform deep matt red colour and showed no melanotic spotting or stippling. The tumour measured approximately 8 mm. × 8 mm. × 5 mm., and was situated on the ventral and ventrolateral surfaces in the region of the gonapodium, which was ensheathed by the tumour mass (Fig. 1, 2). The colour of the tumour was a deep scarlet and the surface coarsely lobular. On section the tumour was a dark red firm mass which encircled the body of the fish ventrally and ventrolaterally, and extended deeply into the muscle mass (Fig. 3). No metastases were observed. The scales around and over the tumour were thickened and appeared fleshy.

*Microscopical appearance.*—The cytological elements of this tumour show considerable pleomorphism. At the periphery the tumour consisted mainly of small and large stellate cells with small nuclei and abundant vacuolated cytoplasm (Fig. 4, 5). The central parts of the growth showed a more bizarre pattern with fusiform cells, round cells, stellate cells and multinucleated forms (Fig. 6).

EXPLANATION OF PLATES.

FIG. 1.—Left lateral view of the red platyfish showing tumour mass, ensheathed gonapodium, ventral fins and scales. × 3·5.

FIG. 2.—Ventral view of same fish. Note large size of tumour. × 3·5.

FIG. 3.—Transverse section of fish at level of gonapodium showing extensive infiltration of the ventral muscle mass, gonapodium and scales. × 6.

FIG. 4.—Peripheral zone of tumour showing stellate cells. Note intact epithelium on surface of tumour. H. & E. × 84.

FIG. 5.—Stellate cells and fine pigment granules. H. & E. × 300.

FIG. 6.—Area of tumour showing multinucleate cells. H. & E. × 135.

FIG. 7.—Unstained frozen section showing birefringent material in tumour cells. × 100.

FIG. 8.—Scales embedded in tumour cells. H. & E. × 55.

FIG. 9.—Hyperplastic erythrophoretic tissue (erythrosis) distant from tumour site. H. & E. × 55.

FIG. 10.—Invasion of striated muscle. H. & E. × 270.

1

2

3

4

5

Ghadially and Whiteley.

6 7 8 9 10

Ghadially and Whiteley.

There was also considerable variation in the size and shape of nuclei, varying from the large vesicular with chromatin granules condensed on the nuclear membrane to small dark pyknotic type with no obvious chromatin pattern. Mitotic figures were extremely scanty.

In haematoxylin and eosin sections the cytoplasm had a foamy vacuolated appearance, in many places obscuring the cell outline. Most of the cells contained a very fine granular brownish-red pigment in varying amounts. Unstained frozen sections showed numerous fine yellow to orange lipoid droplets in the cytoplasm, and these had a tendency to a perinuclear arrangement. The colour of this lipoid material faded almost completely on keeping. Birefringent material was present in many of these cells (Fig. 7).

The carnous appearance of the gonapodium, ventral fins and the scales adjacent to the tumour was found to be due to the embedding of these structures in thick sheets of tumour cells (Fig. 8). This change was also present, but to a lesser degree, in scales distant from the tumour (Fig. 9). There was extensive destructive infiltration of the underlying musculature (Fig. 3, 10), involving a large part of the ventral muscle mass.

### DISCUSSION.

Apart from the few naturally occurring erythrophoromas, the only other reported tumours have been produced by genetic methods in *P. maculatus* × *X. hellerii* hybrids (Nigrelli, Jakowska and Gordon, 1951 ; Smith, Coates and Strong, 1936 ; Kosswig, 1929, 1931). Nigrelli, Jakowska and Gordon (1951) find that xanthoerythrophoromas (tumours similar to the one described in this paper) and melanomas occur in the platyfish-swordtail hybrid due to the modifying influence of the swordtail genes on the colour-producing genes in the male ruby-throated spot-sided platyfish, and further that these tumours occur only in hybrids which have both erythrophores and macromelanophores.

However, the evidence presented in this paper shows that erythrophoromas can occur in the platyfish in the absence both of the modifying swordtail genes and melanophores, as there is no evidence to suggest that our specimen is a hybrid, nor were any spots or stipples present on this fish or other fish from this strain so far examined. Histological examination confirmed the absence of melanin-containing cells in the corium of the fish and tumour.

It will be observed (Fig. 9) that there was hyperplasia of the erythrophoretic tissue distant from the tumour mass, and that this did not show the pleomorphism of the main tumour mass, but was composed of almost normal-looking erythrophores. It is possible, therefore, that this does not represent an invasion of tumour cells in the corium of the fish, but a state of hyperplasia, possibly genetically determined. This is further supported by the fact that we have observed varying grades of hyperplasia in other tumour-free fish from this strain. It seems therefore that this tumour has arisen from a large field of genetically determined hyperplastic tissue, and enlarged not only by cellular proliferation and invasion, but also by progressive neoplastic transformation of hyperplastic tissues within these areas. Thus the genetic constitution of these fish provides susceptible, unstable pre-cancerous areas of tissue which may undergo a malignant change *in situ*. This hyperplastic state, with or without an obvious tumour, may be termed erythrosis, and is analogous to the varying grades of melanosis described by Reed and Gordon (1931) in the platyfish-swordtail hybrids.

SUMMARY.

A case of an invasive red-pigmented tumour (erythrophoroma) has been described in a pure line red platyfish (*P. maculatus* var. *rubra*). This was a rapidly growing tumour which was locally invasive but showed no metastasis. It would appear that this tumour had arisen in a pre-existing genetically-determined bed of hyperplastic tissue (erythrosis).

This is the first record of an erythrophoroma in a pure line platyfish, all others having been reported in platyfish-swordtail hybrids or other species. Thus it would seem that though the modifying swordtail genes described by Nigrelli, Jakowska and Gordon (1951) may be a factor in tumour production in the hybrid, their absence does not preclude the development of tumours in the pure line platyfish.

We would like to thank Professor H. N. Green for valuable advice and criticism. The tumour occurred in a home aquarium kept by Mr. D. J. Wallace, and we are deeply grateful to him for the opportunity of examining the specimen and for his observations on the natural history of the disease. We are indebted to the breeder, Mr. J. D. L. Fleetwood, for supplying the genetic history of the strain, and to Mr. A. W. Collins for the photomicrographs.

Since describing the above specimen a histologically identical tumour, approximately ½ cm. in diameter, invading the muscles has developed in another one of the 4 male red platyfish.

REFERENCES.

GORDON, M.—(1937) *Amer. J. Cancer*, **30**, 362.—(1948) " The Biology of Melanomas," *Spec. Publ. New York Acad. Sci.*, **4**, 216.
JOHNSTONE, J.—(1925) *Proc. Lpool. biol. Soc.*, **39**, 169.
KOSSWIG, C.—(1929) *Z. indukt. Abstamm. Vererb.*, **52**, 114.—(1931) *Ibid.*, **59**, 61.
NIGRELLI, R. F., JAKOWSKA, S., AND GORDON, M.—(1951) *Brit. J. Cancer*, **5**, 54.
REED, H. D., AND GORDON, M.—(1931) *Amer. J. Cancer*, **15**, 1524.
SCHROEDERS, V. D.—(1908). Quoted by Schlumberger, H. G., and Lucke, B., (1948) *Cancer Res.*, **8**, 657.
SMITH, G. M.—(1934) *Amer. J. Cancer*, **21**, 596.
*Idem*, COATES, C. W., AND STRONG, L. C.—(1936) *Zoologica, N.Y.*, **21**, 219.
TAKAHASHI, K.—(1929) *Z. Krebsforsch*, **29**, 1.
THOMAS, L.—(1931) *Bull. Ass. franç. Cancer*, **20**, 703.